# The dual use of traditional cigarettes and e-cigarettes and behavioral and psychological associated factors among Chinese adolescents

**DOI:** 10.1186/s13690-026-02032-9

**Published:** 2026-08-04

**Authors:** Kaiyuan Min, Runping Ma, Lewen Fu, Ziyan Meng, Qing Li, Juntao Yang

**Affiliations:** 1 State Key Laboratory of Common Mechanism Research for Major Diseases, Institute of Basic Medical Sciences & School of Basic Medicine, Chinese Academy of Medical Sciences & Peking Union Medical College, Beijing, China; 2 School of Health Policy and Management, Chinese Academy of Medical Sciences & Peking Union Medical College, Beijing, China

**Keywords:** Cigarettes, E-cigarettes, Electronic nicotine delivery systems, Current Dual use, Adolescents

## Abstract

**Background:**

The dual use of traditional cigarettes and e-cigarettes is common among adolescents. Dual use is likely more harmful than smoking traditional cigarettes or using e-cigarettes alone. However, few studies have explored the behavioral and psychological factors associated with this pattern of tobacco use among Chinese adolescents.

**Methods:**

We surveyed high school students in all 31 provincial-level administrative divisions (PLADs) in mainland China. Tobacco use was categorized as current dual use, exclusive traditional cigarette use, exclusive e-cigarette use, and non-use. We collected 11 behavioral and psychological variables potentially associated with current tobacco use, including physical activity, screen time, sleep duration, diet, alcohol use, fighting, social self-control, self-esteem, self-efficacy, stress, and depression. Multinomial logistic regression adjusted for PLAD fixed effects was employed to analyze the associations.

**Results:**

The weighted sample included 15,000 students with an equal distribution of sex and a median age of 17 years (IQR: 16–18)**.** About 6.9% (95% confidence interval: 6.5%–7.3%) reported current dual use. Several behavioral and psychological factors, including excessive screen time (OR = 0.70, 95% CI = 0.61–0.80; appropriate screen time as the reference group), unhealthy dietary habits (OR = 1.34, 95% CI = 1.15–1.56; healthy diet as the reference group), alcohol use (occasional: OR = 5.53, 95% CI = 4.27–7.17; habitual: OR = 12.71, 95% CI = 9.24–17.47; never use as the reference group), fighting (occasionally: OR = 1.69, 95% CI = 1.41–2.03; habitual: OR = 1.83, 95% CI = 1.18–2.82; never fight as the reference group), strong social self-control (OR = 0.69, 95% CI = 0.57–0.82; moderate as the reference group), strong self-esteem (OR = 1.62, 95% CI = 1.36–1.94; moderate as the reference group), self-efficacy (strong: OR = 1.61, 95% CI = 1.38–1.88; weak: OR = 0.78, 95% CI = 0.62–0.97; moderate as the reference group), and strong stress (OR = 0.78, 95% CI = 0.64–0.95; moderate as the reference group), were found to be associated with the current dual use. Some of the above factors were also associated with the current exclusive use of traditional cigarettes or e-cigarettes.

**Conclusions:**

This study reports on the phenomenon of current dual use and its behavioral and psychological associated factors among high school students in China. Families and schools should pay attention to adolescents’ health risk behaviors and psychological health, as these factors were found to be associated with tobacco use.

**Clinical trial number:**

Not applicable.

**Supplementary Information:**

The online version contains supplementary material available at https://doi.org/10.1186/s13690-026-02032-9.

**Table:** 

Text box 1. Contributions to the literature
This study offers nationally representative evidence on the dual use of cigarettes and e-cigarettes among adolescents in China.
It extends existing knowledge by investigating the roles of behavioral and psychological factors in adolescents’ dual use of cigarettes and e-cigarettes.
The findings provide empirical support for designing public health policies and interventions to prevent adolescent tobacco use in China and other countries with comparable socioeconomic and regulatory backgrounds.

## Introduction

Tobacco use during adolescence is a significant global public health concern with profound health implications. Most adult smokers begin smoking during childhood or adolescence. Compared with adults, adolescents can develop nicotine dependence more rapidly and with fewer cigarettes [1]. In recent years, e-cigarettes have been very popular among adolescents. Evidence from international surveys suggests that e-cigarette use among adolescents is widespread across multiple world regions. Data from the 2022 Health Behaviour in School-aged Children (HBSC) survey showed that, on average, 19.4% of 15-year-olds across participating European countries and Canada reported using e-cigarettes in the past 30 days, with prevalence exceeding 30% in some countries [2]. The Global Youth Tobacco Survey (GYTS) has further demonstrated that past-30-day e-cigarette use among youths reached a global prevalence of 9.2% [3]. In mainland China, 9.5% of high school students were e-cigarette users [4], and the prevalence of e-cigarette use among adolescents is higher than that in other age groups [5]. However, the use of e-cigarettes has not led adolescents to completely give up traditional cigarettes. Studies have recently shown that the use of multiple tobacco products, particularly the dual use of traditional cigarettes and e-cigarettes, is common among adolescents. A survey conducted in 2020 revealed that 38.9% of American youth who were current e-cigarette users also used other tobacco products and that the dual use of e-cigarettes and combustible tobacco was the most common pattern [6]. Similarly, a study in China reported that 48.3% of college students who were tobacco users were dual users [7].

E-cigarette users are exposed to a different mixture of toxic chemicals than those found in traditional cigarettes, including compounds that are generated during the heating and aerosolization processes but are not present in the original e-liquid. As a result, dual users are exposed to a broader and more complex range of toxic substances than those who exclusively use either e-cigarettes or traditional cigarettes [8]. Therefore, due to the significant public health impact of dual use, it is important to better understand the factors associated with combined tobacco and nicotine use patterns to inform the most appropriate and effective public health responses.

Most previous studies have focused on single tobacco products, with limited research on the dual use of traditional cigarettes and e-cigarettes among adolescents or how the associations between dual use and various factors differ from the use of single products [6, 9, 10]. Some research on dual use has focused on social‒environmental factors, such as peer influence, family relationships, and advertising exposure [11–13], with relatively less attention given to individual behavioral and psychological factors. However, behavioral and psychological factors play critical roles in health across the lifespan [14]. Health risk behaviors (HRBs), such as unhealthy eating habits, insufficient physical activity, and tobacco use, are prevalent during adolescence and seldom occur in isolation [15, 16]. Therefore, when analyzing the associated factors for dual use, it is essential to recognize the associations of dual use with other health risk behaviors to develop more effective health promotion and prevention strategies [17].

In addition, the dual use of e-cigarettes and traditional cigarettes may be associated with psychological factors. Previous studies have demonstrated that psychological symptoms, such as stress and depression, predict adolescent smoking [18]. Low self-esteem and self-control are associated with a greater risk of smoking [19, 20]. Self-efficacy, defined as individuals’ beliefs about their ability to exercise control over their actions and associated outcomes, is also a risk factor for adolescent smoking [21].

Moreover, China is the world’s largest consumer of tobacco products and contributes substantially to the global burden of smoking-related diseases [22]. However, previous research on dual use has been conducted mainly in Europe and the United States, with limited evidence from China. China ratified the World Health Organization (WHO) Framework Convention on Tobacco Control (FCTC) in 2005, and the treaty came into force in the country on January 9, 2006. Although China has made notable progress in tobacco control since then, substantial gaps remain in fully meeting the FCTC requirements [23]. For instance, tobacco taxation remains relatively low, and both package warnings and Smoke and Aerosol-Free Environment (SAFE) environment policies in China are considerably weaker compared to those in Hong Kong and many other regions [24]. Additionally, China has the second-largest adolescent population in the world, accounting for 12.9% of the global adolescent population [25]. Therefore, researching the factors associated with current dual use among Chinese adolescents is both meaningful and urgent.

To fill these gaps, this study aims to examine the prevalence of different tobacco use patterns and the associations between behavioral and psychological factors and current dual use of traditional cigarettes and e-cigarettes among adolescents in China. This study seeks to enhance understanding of adolescent tobacco and nicotine use patterns and to inform the development of more targeted prevention strategies in China and other sociocultural contexts.

## Methods

### Survey design

We conducted an anonymous online cross-sectional survey using a two-step nationwide design, administered by KuRunData, a platform that has been extensively utilized in previous research for data collection [26, 27]. The survey was conducted between July and September 2021. Before the formal survey, a pilot study involving 104 high school students was conducted to refine the questionnaire and estimate the sample size. We used the sample size calculator developed by Wang et al. [28] to estimate the minimum required sample size. Based on the results of an online pilot survey, we set the proportion of current e-cigarette users among high school students (*p*) to 8.7%. We set the type I error rate (*α*) at 0.05 and the absolute error (*d*) at 0.5%. With these parameters, the minimum sample size was estimated to be 12,206. To simplify the sample size allocation process, we increased the overall sample size goal to 15,000.

In the first stage, the overall sample size goal was allocated to each of 31 provincial-level administrative divisions (PLADs) based on the proportion of high school students in each PLAD relative to the national total [29, 30]. In the second stage, we stratified the sampling within each PLAD by sex (male or female) [30], residence (urban or rural) [31], and school type (general high schools or vocational high schools) to obtain representative samples [29, 30]. We further allocated sample size goals to each stratum based on the proportion of high school students in each stratum relative to the total number in the corresponding PLAD. To minimize sampling errors, we oversampled strata with sample size goals below 30 to ensure that at least 30 students were surveyed per stratum. This resulted in the actual number of surveyed students exceeding the predetermined goal of 15,000, reaching 16,365 participants.

Considering that high school students may not have internet access during academic semesters, the survey was conducted during the summer break from July to September 2021. A total of 21,085 high school students accessed the e-questionnaire, and 17,539 completed it. Of the 17,539 responses, 613 were determined to be ineligible, and 561 were kept as backup data to avoid inadequate sample sizes caused by cleaning ineligible data. The 561 backup responses resulted from a technical delay in closing the online survey after the regional target sample sizes had been reached. Specifically, responses were classified as backup if they were submitted after the predefined target sample size for a given stratum had already been achieved. However, these 561 responses were not included in the final analysis. Ultimately, 16,365 respondents were included in our analysis.

To ensure that the composition of our sample aligned with official statistics for each PLAD in terms of sex, residence, and educational attainment, we assigned sample weights to each respondent [29–31]. These weights were calculated by dividing the sample size goal by the actual number of respondents within each stratum. This weighting approach enables our data to be nationally representative**.** Comprehensive details regarding the survey implementation and quality control procedures are provided in the Supplementary Methods.

### Measurement

#### Outcome variable: tobacco use status

Tobacco use status was the outcome variable in our analysis. This variable was classified into four categories: current exclusive use of traditional cigarettes, exclusive use of e-cigarettes, dual use, and non-use of either e-cigarettes or traditional cigarettes. Based on previous research, the use of traditional cigarettes and e-cigarettes was determined by the self-reported frequency of use in the past 30 days. Any response indicating use on one or more days was considered current use [5, 32]. Respondents who reported current use of traditional cigarettes but did not use e-cigarettes were classified as current exclusive traditional cigarette users, whereas those who used only e-cigarettes and did not use traditional cigarettes were classified as current exclusive e-cigarette users. Individuals who had used both traditional cigarettes and e-cigarettes in the past 30 days were classified as current dual users, whereas those who had not used either traditional cigarettes or e-cigarettes were classified as current non-users.

#### Behavioral and psychological factors potentially associated with current tobacco use status

Based on previous studies [15, 16, 18–21], we collected data on 11 factors potentially associated with the current dual use of traditional cigarettes and e-cigarettes, including six behavioral and five psychological factors. Behavioral factors (physical activity, diet, sleep duration, screen time, alcohol use, and fighting) were included because they are modifiable lifestyle behaviors that can be targeted in preventive interventions. Psychological factors (social self-control, self-esteem, self-efficacy, stress, and depression) were examined as they may serve as indicators to identify adolescents at risk of engaging in risky behaviors.

Physical activity was assessed using items adapted from the International Physical Activity Questionnaire (IPAQ) [33]. Diet, alcohol use, and fighting were adapted from the Global School-based Student Health Survey (GSHS) core questionnaire modules [34]. Social self-control was measured using the Chinese version of the Social Self-Control Scale (Cronbach’s α = 0.76 in this study) [35]. Self-esteem was assessed using the Chinese version of the Rosenberg Self-Esteem Scale (Cronbach’s α = 0.90) [36]. Self-efficacy was measured using the Chinese version of the General Self-Efficacy Scale (Cronbach’s α = 0.88) [37]. Stress was measured using the Chinese version of the 10-item Perceived Stress Scale (PSS-10) (Cronbach’s α = 0.71) [38], and depression was assessed using the Chinese version of the Patient Health Questionnaire (PHQ-9) (Cronbach’s α = 0.87) [39], Other items were self-developed for this study. Given that psychological scale scores may not exhibit a strictly linear relationship with the outcome, these variables were treated as categorical variables in the regression analyses. The specific details can be found in Table S1.

#### Covariates

The following control variables were included in the analysis: age (years), sex (male, female), current parental tobacco use, defined as current use of traditional cigarettes, e-cigarettes, or both (neither parent, father only, mother only, and both parents), and body mass index (BMI) and its quadratic term (both centered to reduce potential collinearity, with both variables centered by subtracting the mean value), to account for potential non-linear associations between BMI and tobacco use [40–42].

### Data analysis

We used descriptive statistics to summarize the characteristics of the survey participants. We present the numbers and proportions of the sample size for each PLAD. We described sample characteristics including age, sex, residence, school type, and grade. Continuous variables were presented as medians and interquartile ranges (IQRs). Categorical variables were presented as frequencies and percentages. Age was presented as both a categorical variable and a continuous variable. Additionally, we present the prevalence of current traditional cigarette use, e-cigarette use, and dual use among male and female high school students by PLAD using bivariate choropleth maps.

Because our outcome variable was classified into four categories, we used a multinomial logistic regression model to analyze the behavioral and psychological factors associated with current dual use status: exclusive use of traditional cigarettes, exclusive use of e-cigarettes, dual use, and non-use of either e-cigarettes or traditional cigarettes [4]. Compared with other regression models, the multinomial logistic regression model can provide odds ratios for each category compared with the reference category to offer insights into the likelihood of different outcomes. In this multinomial logistic model, current tobacco use status was designated as the outcome variable, with non-use of either e-cigarettes or traditional cigarettes as the reference category, and behavioral and psychological factors were included as exposure variables. Covariates, including age, sex, and centered BMI and its quadratic term, were also included in the model. Considering that some unmeasurable confounders were not included in the model but were consistent within each PLAD, we incorporated binary indicators for PLADs as fixed effects in the model. This methodological approach has been widely used in previous research [27, 43, 44]. The effect sizes of the associations between behavioral and psychological factors were presented as adjusted ORs with 95% confidence intervals (CIs) in a forest plot. Previous studies have reported sex and age group differences in tobacco use [45–48]. Therefore, in addition to performing multinomial logistic regression on the entire sample, we conducted subgroup analyses by sex and age group.

To account for the multistage sampling process in the survey, we performed complex survey data analyses for all statistical inferences of standard errors, 95% CIs, and statistical significance, with standard errors were clustered at the level of primary sampling units [49]. All statistical tests were two-sided, with statistical significance denoted at *P* < 0.05. All data analyses were conducted with Stata Statistical Software version 18 (StataCorp., Texas., USA) [50]. Bivariate choropleth maps and forest plots were generated using R 4.3.3 (R Core Team, 2024) [51].

## Results

### Sample characteristics

In the unweighted sample, the largest proportions of students were from Henan (8.3%), Guangdong (6.8%), and Shandong (6.1%) (Table S2). The weighted sample of 15,000 students included approximately equal proportions of males (49.9%) and females (50.1%). The median age of the students was 17 years (IQR: 16–18). The majority of the students resided in urban areas (59.5%), attended general high schools (66.5%), and were in the 12th grade (48.8%). Few differences were observed in sample characteristics between the unweighted (Table S3) and weighted (Table 1) samples.

**Table 1:** Sample characteristics by e-cigarette and traditional cigarette current use status, weighted

Characteristic^a^	Full sample (n_weighted_ = 15,000; 100%), n (%)	Current dual users (n_weighted_ = 1,029; 6.9%), n (%)	Current exclusive traditional cigarette users (n_weighted_ = 2,655; 17.7%), n (%)	Current exclusive e-cigarette users (n_weighted_ = 398; 2.7%), n (%)	Current non-users (n_weighted_ = 10,918; 72.8%), n (%)
Male, n (%)	7,483 (100%)	874 (11.7%)	2,351 (31.4%)	287 (3.8%)	3,971 (53.1%)
Age, median (Q1, Q3)	17 (16, 18)	18 (17, 18)	17 (17, 18)	17 (16, 18)	17 (16, 18)
Age (years), n (%)					
≤ 16	2,004 (13.4%)	60 (5.8%)	224 (8.4%)	48 (12.1%)	1,672 (15.3%)
16–17	3,709 (24.7%)	207 (20.1%)	629 (23.7%)	109 (27.4%)	2,765 (25.3%)
17–18	5,374 (35.8%)	365 (35.4%)	1,163 (43.8%)	136 (34.1%)	3,711 (34.0%)
> 18	3,914 (26.1%)	398 (38.6%)	639 (24.1%)	105 (26.5%)	2,771 (25.4%)
Urban residence, n (%)	8,924 (59.5%)	697 (67.7%)	1,866 (70.3%)	201 (50.5%)	6,160 (56.4%)
School type, n (%)					
General high schools	9,977 (66.5%)	355 (34.5%)	1,743 (65.6%)	118 (29.6%)	7,762 (71.1%)
Vocational high schools	5,022 (33.5%)	674 (65.5%)	912 (34.4%)	280 (70.4%)	3,156 (28.9%)
Grade, n (%)					
10th	3,069 (20.5%)	130 (12.6%)	382 (14.4%)	81 (20.5%)	2,476 (22.7%)
11th	4,605 (30.7%)	273 (26.6%)	936 (35.3%)	135 (34.0%)	3,260 (29.9%)
12th	7,326 (48.8%)	626 (60.8%)	1,336 (50.3%)	181 (45.5%)	5,182 (47.5%)

The presented results were derived from a weighted sample. ^a^Percentages are presented by column for all categorical variables, except for “male,” for which row percentages are reported to show the distribution of tobacco use status within males

*n (%)* frequency with percentage, *IQR* interquartile range

### Current tobacco use status among Chinese adolescents

In this survey, 6.9% (95% CI: 6.5%–7.3%) of the students in the weighted sample reported current dual use of traditional cigarettes and e-cigarettes. The prevalence of current dual use among male students was 11.7% (95% CI: 11.0%–12.4%), while the prevalence among female students was 2.1% (95% CI: 1.8%–2.4%). Geographically, the highest prevalence of dual use was found in eastern areas such as Shanghai (11.7%, 95% CI: 4.4%–27.8%), Beijing (11.1%, 95% CI: 4.9%–23.4%), and Hainan (9.9%, 95% CI: 4.8%–19.3%). The lowest prevalence was observed in western areas such as Chongqing (4.1%, 95% CI: 1.8%–9.0%), Guangxi (4.2%, 95% CI: 1.5%–11.2%), and Shaanxi (4.9%, 95% CI: 2.4%–9.7%) (Figure S1a, Table S6). Nearly a quarter (24.6%, 95% CI: 23.9%–25.3%) of high school students reported the current use of traditional cigarettes. Among traditional cigarette current users, 27.9% (1,029/3,684) were dual users (Figure S1b, Table S4). Approximately one-tenth (9.5%, 95% CI: 9.1%–10.0%) of high school students used e-cigarettes. Among e-cigarette users, 72.1% (1,029/1,427) were dual users (Figure S1c, Table S5).

### Behavioral and psychological factors associated with current tobacco use status

Several findings from the weighted sample highlight the behavioral and psychological characteristics of high school students. With regard to behavioral factors, we found that, on average, Chinese high school students participate in only 13 min of moderate-to-vigorous physical activity per day. Additionally, 55.7% of high school students spend more than two hours on screens daily, and 67.4% do not get sufficient sleep. Unhealthy diets are also prevalent, with 49.6% of students reporting poor dietary habits. Alcohol use is common, with 69.5% of students consuming alcohol; furthermore, 11.0% of students engage in physical fights. In terms of psychological factors, 11.6% of the students experienced symptoms of moderately severe or severe depression (Table S7).

**Fig. 1:**
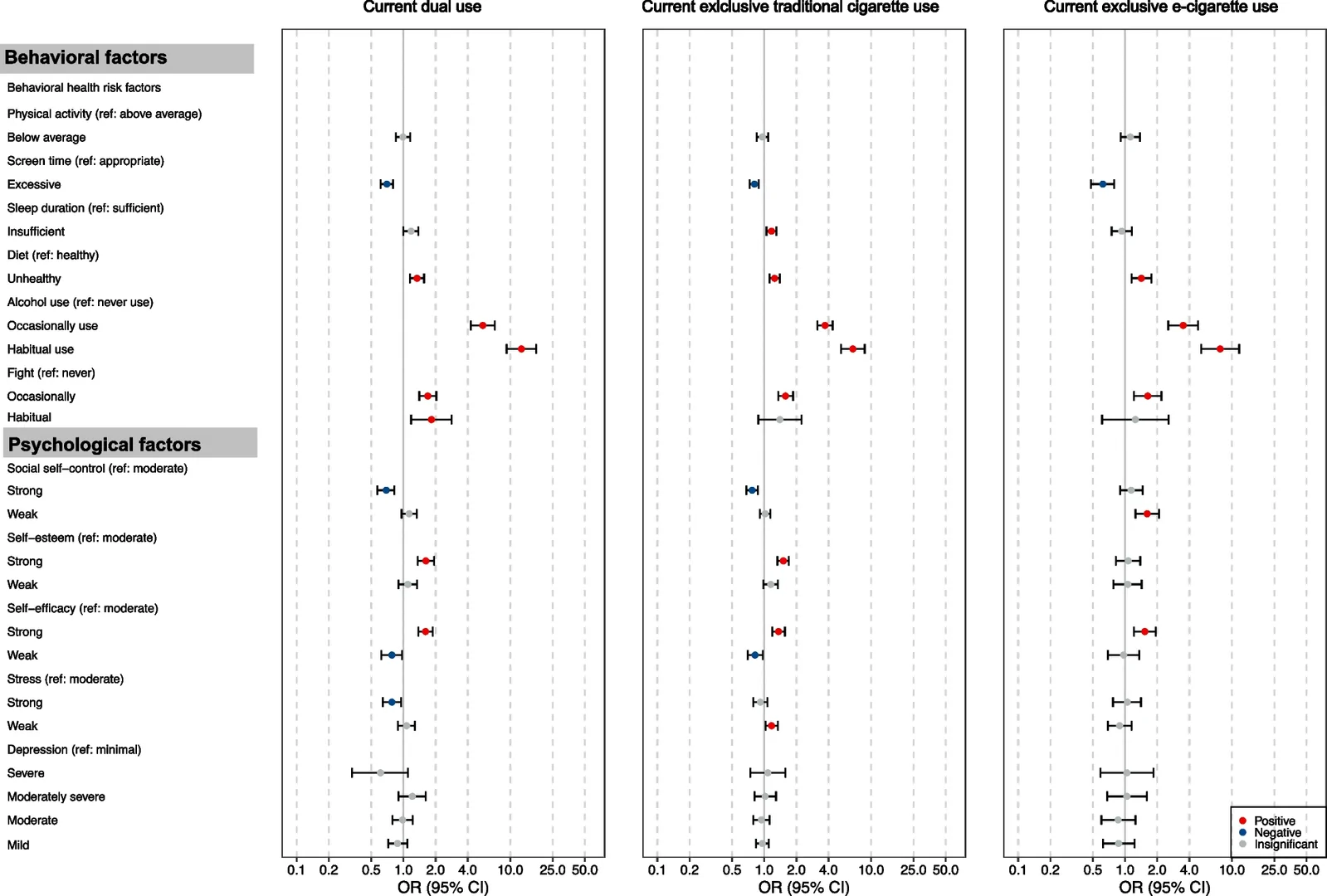
Factors associated with current dual use, exclusive traditional cigarette use, and exclusive e-cigarette use among high school students in China, weighted. The presented results were derived from a weighted mutually adjusted multinomial logistic regression model with current non-use of either e-cigarettes or traditional cigarettes as the reference category. The model controlled for fixed effects of PLADs. The variance inflation factors for the exposure variables ranged from 1.04 to 1.99, indicating no significant multicollinearity [52]. ref, reference; OR, odds ratio; CI, confidence interval; PLAD, provincial-level administrative division

A weighted multinomial logistic regression model revealed that most behavioral factors were associated with current dual-use status. Among the behavioral factors, excessive screen time (OR = 0.70, 95% CI: 0.61–0.80; appropriate screen time as the reference group) was inversely associated with dual use, whereas unhealthy diet (OR = 1.34, 95% CI: 1.15–1.56; healthy diet as the reference group), alcohol use (occasional: OR = 5.53, 95% CI: 4.27–7.17; habitual: OR = 12.71, 95% CI: 9.24–17.47; never use as the reference group), and fighting (occasional: OR = 1.69, 95% CI: 1.41–2.03; habitual: OR = 1.83, 95% CI: 1.18–2.82; never fight as the reference group) were positively associated with dual use. Among the psychological factors, when moderate levels were used as the reference, strong self-esteem (OR = 1.62, 95% CI: 1.36–1.94; moderate as the reference group), and strong self-efficacy (OR = 1.61, 95% CI: 1.38–1.88; moderate as the reference group) were positively associated with dual use, whereas strong social self-control (OR = 0.69, 95% CI: 0.57–0.82; moderate as the reference group), strong stress (OR = 0.78, 95% CI: 0.64–0.95; moderate as the reference group) and weak self-efficacy (OR = 0.78, 95% CI: 0.62–0.97; moderate as the reference group) was associated with a lower likelihood of dual use (Fig. 1, Table S8).

Many of the significant factors mentioned above were also associated with the current exclusive use of traditional cigarettes and e-cigarettes, with some exceptions. Habitual fighting was not associated with the exclusive use of traditional cigarettes despite being linked to dual use. In contrast, although insufficient sleep was not associated with dual use, it was associated with the exclusive use of traditional cigarettes. Weak stress (compared with moderate stress: OR = 1.17, 95% CI: 1.03–1.34) and weak self-efficacy were (compared with moderate self-efficacy: OR = 0.82, 95% CI: 0.70–0.97) associated with the exclusive use of traditional cigarettes but not with dual use. Additionally, insufficient sleep duration, habitual fighting, strong social self-control, strong self-esteem, weak self-efficacy, and stress were not associated with exclusive e-cigarette use. Weak social self-control (OR = 1.61, 95% CI: 1.25–2.08; moderate as the reference group) was associated with exclusive e-cigarette use but was not linked to dual use (Fig. 1, Table S8).

No apparent heterogeneity was observed when the analyses were stratified by sex and age group. For specific details, please refer to Table S9 and Table S10.

## Discussion

In this study of a nationally representative, weighted sample of 15,000 Chinese high school students, we examined behavioral and psychological factors associated with current tobacco use. We found that over one-quarter of students used traditional cigarettes, about one-tenth used e-cigarettes, and 6.9% were dual users. Several behavioral and psychological factors—including screen time, diet, alcohol use, fighting, and self-regulation measures—were associated with dual use, with similar patterns observed for exclusive use. These findings underscore the importance of addressing adolescents’ health behaviors and psychological well-being in efforts to reduce tobacco use.

The proportion of dual users among the high school students in this study (6.9%) is comparable to the percentage reported among adolescents in Canada (6.3%) [53]. A previous study revealed that the prevalence of dual use in 67 countries ranged from 0.1% to 13.6%, and the prevalence of dual use exceeding 5% in only 15 countries. This suggests a high prevalence of dual use among Chinese adolescents [54]. Considering that 27.9% of traditional cigarette users and 72.1% of e-cigarette users are dual users, interventions that target either e-cigarette use or traditional tobacco use exclusively may not be highly effective because a significant number of adolescents are dual users. Therefore, more comprehensive intervention strategies that target the dual use of e-cigarettes and traditional cigarettes are needed.

We found that most behavioral factors were associated with dual use. Unhealthy diets, alcohol consumption, and fighting were all associated with a higher risk of dual use, reflecting the clustering of unhealthy behaviors. In particular, alcohol use was associated with greater risk of dual use among adolescents, which is consistent with the findings of previous studies [55]. These findings suggest that adolescents' health risk behaviors are closely related and that these behaviors should be regarded as associated indicators of traditional cigarette and e-cigarette use to enable early detection and timely intervention.

However, adolescents with more than two hours of daily screen time showed a lower likelihood of dual use, differing from prior studies [56, 57]. This finding may be explained by several context-specific factors. First, the survey was conducted during the COVID-19 pandemic, when China implemented the “Classes Suspended but Learning Continues” emergency plan, requiring all teaching and learning activities to move online [58]. As a result, much of the extended screen time was likely devoted to educational purposes — a type of screen use that existing evidence indicates is associated with lower odds of smoking, in contrast to social or entertainment-related screen activities, which tend to increase smoking risk [59, 60]. Second, China has implemented strict regulations on tobacco-related online content that prohibit direct and indirect forms of advertising including converting advertisements by influencers, films and other forms, aiming to limit adolescents’ exposure to tobacco-related content [24, 61, 62]. These considerations support the plausibility of our finding that longer screen time was associated with lower dual use risk and underscore the need for future research to differentiate types of screen activities when examining their relationship with adolescent tobacco use.

Given that the behavioral factors associated with dual use—such as alcohol use, fighting, and cigarette use—are strongly interconnected, preventing the dual use of e-cigarettes and traditional cigarettes among adolescents requires comprehensive interventions that target multiple health risk behaviors simultaneously, rather than focusing on a single behavior [63]. Accordingly, several strategies can be implemented. First, parents can be supported through structured family-based interventions delivered or reinforced by schools and public health programs, which provide guidance on healthy lifestyle practices and parenting skills. By improving parents’ own health literacy and behavioral modeling, such programs may help create a supportive home environment that reduces the likelihood of cigarette and e-cigarette use among adolescents [64–66]. Second, schools and families should strengthen collaboration, particularly for adolescents who exhibit behaviors such as tobacco use, alcohol consumption, or fighting, as these behaviors are closely linked to dual use. Close monitoring and timely intervention are therefore essential [67]. Evidence from existing programs supports this integrated approach. For example, the Adolescent Transition Program in middle schools in the United States combines a universal classroom-based intervention, the Family Check-Up, and family management treatments to target multiple problem behaviors simultaneously [68]. Similarly, the Family-School Partnership (FSP) program in the United States, which involves weekly home-school learning sessions, communication activities, and periodic parenting workshops, has demonstrated that coordinated efforts between families and schools can effectively reduce the risk of tobacco smoking [69].

Among the psychological factors, firstly, the observed association between social self-control and dual use supports previous evidence, highlighting the role of self-regulation in adolescents’ susceptibility to tobacco use [70]. Adolescents with poor social self-control are more likely to associate with deviant peers who are more likely to use substances, making them more susceptible to smoking under peer influence [71, 72]. Secondly, adolescents with higher self-esteem and self-efficacy were more likely to engage in dual use, contrary to previous findings [73]. This may be explained by the fact that individuals with low self-esteem may lack confidence in their abilities and avoid risky behaviors such as smoking, whereas those with high self-esteem may have an elevated sense of their capabilities, increasing their likelihood of engaging in risky behaviors [74]. Similarly, individuals with strong self-efficacy tend to have greater confidence in their ability and may believe that they can quit anytime or manage the negative consequences [75–77]. Finally, we found that higher stress were associated with a lower risk of dual use. In the Chinese context, adolescents’ stress primarily stems from intense academic demands, which are reinforced by stronger parental and teacher expectations compared with those faced by their peers in many Western countries [78, 79]. Those tend to increase students’ compliance with school rules and reduce the amount of free time and social opportunities available for engaging in risky behaviors such as smoking. As a result, academically driven stress may inadvertently limit adolescents’ likelihood of dual use.

Based on the observed associations between psychological factors and dual use, prevention initiatives may consider integrating psychological skill development into tobacco control programs [80]. For example, the Life Skills Training Program in New York State integrates cognitive-behavioral skill development, including strengthening self-esteem, resisting advertising influence, managing anxiety, and improving communication, which provides a valuable model for future interventions [81]. In addition, the European “Unplugged” program, evaluated in the EU-Dap cluster randomized controlled trial, incorporates social and emotional skill training, normative education, and critical thinking components to prevent tobacco and other substance use among adolescents, and has demonstrated effectiveness in reducing smoking initiation in randomized controlled trials, further supporting the value of psychological skill–based approaches in tobacco prevention [82].

Based on our findings and previously validated interventions, we recommend that China’s education authorities and other relevant government agencies adopt comprehensive strategies that simultaneously address behavioral and psychological factors associated with the use of traditional cigarettes and e-cigarettes to develop more effective interventions.

Our study has several strengths. First, it included a weighted sample of 15,000 participants from across all 31 PLADs in mainland China, making it both large and nationally representative. Second, the study investigated behavioral and psychological factors associated with current dual use of e-cigarettes and traditional cigarettes and compared them with those among exclusive users. This study, therefore, deepens the understanding of dual use as a specific pattern of tobacco use and provides some empirical evidence regarding associated factors of dual use. Finally, since each province has its own unique policies and cultural contexts for tobacco that are difficult to measure, we included binary indicators of PLADs as fixed effects in the model to effectively control for unmeasurable confounding factors [44].

Our study also has certain limitations. Firstly, it relied on data from an online survey of high school students in China, which limits the extent to which our findings can be generalized to other groups, such as adolescents without internet access or those in different countries. Secondly, among the 21,085 individuals who accessed the questionnaire, only 16,365 were included in the data analysis, with 4,720 excluded. This exclusion may have introduced potential bias into our study findings. Thirdly, because the data were self-reported, there may be biases due to the participants’ inaccurate recall, as well as possible underreporting or overreporting. Finally, the cross-sectional design of our study means that our findings should be viewed as associations rather than causal relationships. It is possible that some of the observed associations reflect reverse causation — adolescents who engage in dual use may subsequently report higher levels of self-esteem or self-efficacy due to perceived social acceptance, autonomy, or a sense of maturity gained from engaging in adult-like behaviors [75–77]. Future studies are encouraged to investigate the causal links between health risk behaviors, psychological factors, and dual-use in prospective cohort studies.

In conclusion, among this large, nationally representative sample, we found that more than one-quarter of students used traditional cigarettes, about one-tenth used e-cigarettes, and 6.9% were current dual users. Dual use was associated with multiple behavioral and psychological factors, including screen time, diet, alcohol use, fighting, social self-control, self-esteem, self-efficacy, and stress, many of which were also related to exclusive use of either product. These findings underscore the importance of family- and school-based efforts to monitor and support adolescents’ health behaviors and psychological well-being to address the growing challenge of dual tobacco and nicotine use.

## Supplementary Information

Supplementary Material 1.

## Data Availability

The data and analytic codes used in this study will be available six months after the time of publication, with no end date, from the corresponding authors upon reasonable request.

## Abbreviations

PLADs: Provincial-level administrative divisions
OR: Odds ratio
CI: Confidence intervals
IQR: Interquartile range
HRB: Health risk behavior
WHO: World Health Organization
FCTC: Framework Convention on Tobacco Control
BMI: Body mass index
