# Supplementary material for: The dual use of traditional cigarettes and e-cigarettes and behavioral and psychological associated factors among Chinese adolescents
Source: Arch Public Health. 2026 Aug 4;84:230. doi: 10.1186/s13690-026-02032-9 (PMC13721673; doi:10.1186/s13690-026-02032-9)
Supplement: Supplementary file 1 [file 13690_2026_2032_MOESM1_ESM.docx]

**Contents**

[**SUPPLEMENTARY METHODS**	2](#_Toc236408047)

[Implementation and quality control of the online survey	2](#_Toc236408048)

[**SUPPLEMENTARY FIGURE**	4](#_Toc236408049)

[Figure S1. Study population	4](#_Toc236408050)

[Figure S2. Prevalence of current dual use, traditional cigarette use, and e-cigarette use among male and female high school students in China by provincial-level administrative division, weighted	4](#_Toc236408051)

[**SUPPLEMENTARY TABLES**	6](#_Toc236408052)

[Table S1. Measurement and categorization of behavioral and psychological factors potentially associated with traditional cigarette use and e-cigarette use status.	6](#_Toc236408053)

[Table S2. Sample sizes by provincial-level administrative divisions	8](#_Toc236408054)

[Table S3. Sample characteristics by traditional cigarette and e-cigarette use status, unweighted	9](#_Toc236408055)

[Table S4. Prevalence of current traditional cigarette use among male and female high school students in China by provincial-level administrative division, weighted	10](#_Toc236408056)

[Table S5. Prevalence of current e-cigarette use among male and female high school students in China by provincial-level administrative division, weighted	11](#_Toc236408057)

[Table S6. Prevalence of current dual use among male and female high school students in China by provincial-level administrative division, weighted	12](#_Toc236408058)

[Table S7. Factors potentially associated with current dual use, exclusive traditional cigarette use and exclusive e-cigarette use among high school students in China, weighted	14](#_Toc236408059)

[Table S8. Factors associated with current dual use, exclusive traditional cigarette use and exclusive e-cigarette use among high school students in China, weighted	16](#_Toc236408060)

[Table S9. Sex subgroup analysis of factors associated with current dual use, exclusive traditional cigarette use and exclusive e-cigarette use among high school students in China, weighted	17](#_Toc236408061)

[Table S10. Age subgroup analysis of factors associated with current dual use, exclusive traditional cigarette use and exclusive e-cigarette use among high school students in China: weighted	20](#_Toc236408062)

[**SUPPLEMENTARY REFERENCES**	24](#_Toc236408063)

**SUPPLEMENTARY METHODS**

**Implementation and quality control of the online survey**

The survey was conducted by KuRunData, an online survey platform that maintains a participant database and administers surveys. KuRunData recruits members through its platform and partnerships with other websites and encourages registered members to invite new members through WeChat, a popular mobile social network application among high school students in China[1-3]. Respondents were recruited through the registered member networks of KuRunData and its partner companies. To expand the surveys’ reach, registered members were encouraged to invite new members via WeChat, a highly prevalent social networking platform in China. Duplicate registrations were identified and excluded from surveys. Registered members also had to pass authenticity and seriousness tests to be eligible for survey participation, and these tests were repeated at regular intervals of at least every 180 days. Basic personal information, including sex, date of birth, education, and location, was collected from registered members. To ensure the accuracy of the information, IP addresses were used to verify the location, and phone numbers and email addresses had to be activated. Additionally, sex and date of birth could not be changed after submission, and changes to the location information required re-verification through the IP address. As an incentive for participation, each respondent who completed the questionnaire received a monetary reward of approximately 15 RMB (2.3 USD), which was automatically transferred to their registered KuRunData account upon successful submission.

Students in the survey pool were invited to participate in the survey through the KuRunData platform. The e-questionnaire used in the survey was created in standard Chinese and hosted on the survey platform of KuRunData. The informed consent page described the project’s background and purpose, possible risks, payment after completing the questionnaire, and the confidentiality of the information and records. Before the students completed the questionnaire, they were required to read the informed consent form for at least 15 seconds and provide their electronic signature to indicate their understanding of the survey’s objectives as well as the potential benefits and risks associated with participation.

The survey platform ensured quality control by requiring the respondents to complete each question before proceeding to the next. The survey platform incorporated automatic logic checks and skipping. For example, if a respondent was identified as a current non-users of e-cigarettes, questions about current e-cigarette use were automatically skipped. In addition, the survey platform instantly excluded respondents who completed the e-questionnaire in less than the preset threshold of five minutes because data from respondents who did not spend sufficient time reading and comprehending the questions would not meet the required quality standards.

**SUPPLEMENTARY FIGURE**

**Figure S1. Study population**

**Figure S2. Prevalence of current dual use, traditional cigarette use, and e-cigarette use among male and female high school students in China by provincial-level administrative division, weighted**

The blank areas represent provincial-level administrative divisions not included in the survey. The presented results were derived from a weighted sample. This map was generated using publicly available data from the Tianditu Cloud Platform (https://cloudcenter.tianditu.gov.cn/administrativeDivision/) under approval number GS(2024)0650.

**SUPPLEMENTARY TABLES**

**Table S1. Measurement and categorization of behavioral and psychological factors potentially associated with traditional cigarette use and e-cigarette use status.**

| **Factor** | **Measurement** | **Categorization** |
| --- | --- | --- |
| Tobacco use | Q1: Have you ever tried or experimented with cigarette use, even one or two puffs?[4]  Q2: During the past 30 days, how many days did you use cigarettes?[4]  Q3: Have you ever tried or experimented with e-cigarette use, even one or two puffs?[5]  Q4: During the past 30 days, how many days did you use e-cigarettes?[5] | 1) Non-use: “no” to Q1, Q3, and “zero days” to Q2, Q4 2) Exclusive traditional cigarette use: “yes” to Q1, “one day or more” to Q2, “no” to Q3 or “zero days” to Q4 3) Exclusive e-cigarette use: “yes” to Q3, “one day or more” to Q4, “no” to Q1 or “zero days” to Q2 4) Dual use: “yes” to Q1 and “one day or more” to Q2 and “yes” to Q3 and “one day or more” to Q4 |
| Physical activity | Adapted from the International Physical Activity Questionnaire (IPAQ)[6]  Q: Typically, how many days per week do you engage in physical activities (including chores, physical education, etc.) that cause your breathing and heart rate to increase mildly to significantly? On those days, for how many minutes per day? | Responses were classified into two categories based on the average (13):   1) Above average: > 13 minutes 2) Below average: ≤ 13 minutes |
| Screen time | Q: Typically, what is your average daily total hours of watching TV, playing video games, or using cell phones, computers, and other devices with screens? | Responses were classified into two categories:[7]   1) Appropriate: ≤ 2 hours 2) Excessive: >2 hours |
| Sleep duration | Q: Typically, what is the total number of hours of sleep you get per day on average (including naps, etc.)? | Responses were classified into two categories:[8]   1) Sufficient: ≥ 8 hours 2) Insufficient: < 8 hours |
| Alcohol use | Adapted from the Global School-based Student Health Survey (GSHS)[9]  Q: In the last year, have you consumed any alcoholic beverages (e.g., beer, red wine, yellow wine, white wine, etc.)? | Responses were encoded from one (never) to five (always) and classified into three categories:   1) Never use: 1 2) Occasionally use: 2-3 3) Habitually use: 4-5 |
| Fight | Adapted from the Global School-based Student Health Survey (GSHS)[9]  Q: In the last year, have you gotten into any fights? | Responses encoded from one (never) to five (always) and classified into three categories:   1) Never fight: 1 2) Occasionally fight: 2-3 3) Habitually fight: 4-5 |
| Diet | Adapted from the Global School-based Student Health Survey (GSHS)[9]  Q1: In the last year, have you eaten fried, barbecued, puffed-up, Western fast food or any similar types of food?  Q2: In the last year, did you drink any sugary drinks (e.g., milk tea) or carbonated drinks (e.g., cola)? | Responses encoded from one (never) to five (always) and classified into two categories:   1) Healthy: Q1 and Q2 ≤3 2) Unhealthy: Q1 or Q2 >3 |
| Social self-control | A Chinese version of the social self-control scale[10] | Responses to ten items encoded from one (never) to four (always) were summed to calculate a score. This score was classified into three categories by the upper quartile (26) and the lower quartile (22).   1) Strong: < 22 2) Moderate: 22-26 3) Weak: > 26 |
| Self-esteem | A Chinese version of the Rosenberg self-esteem scale[11] | Responses to ten items encoded from one (strongly disagree) to four (strongly agree) were summed to calculate a score. This score was classified into three categories by the upper quartile (30) and the lower quartile (26).   1) Strong: > 30 2) Moderate: 26-30 3) Weak: < 26 |
| Self-efficacy | A Chinese version of the general self-efficacy scale[12] | Responses to ten items encoded from one (not at all true) to four (exactly true) were summed to calculate a score. This score was classified into three categories by the upper quartile (27) and the lower quartile (21).   1) Strong: > 27 2) Moderate: 21-27 3) Weak: < 21 |
| Stress | A Chinese version of the ten-item perceived stress scale (PSS-10)[13] | Among the ten items, items 1, 2, 3, 6, 9, and 10 were negative, and items 4, 5, 7, 8 were positive. Responses to negative items were encoded from zero (never) to four (very often), and responses to positive items were encoded from four (never) to zero (very often). These responses were summed to calculate a score. This score was classified into three categories by the upper quartile (17) and the lower quartile (22).   1) Strong: > 22 2) Moderate: 17-22 3) Weak: < 17 |
| Depression | A Chinese version of the Patient Health Questionnaire (PHQ)-9[14] | Responses to nine items encoded from zero (not at all) to three (nearly every day) were summed to calculate a score. This score was classified into five categories by established thresholds.   1) Severe: ≥ 20 2) Moderately severe: 15-19 3) Moderate: 10-14 4) Mild: 5-9 5) Minimal: ≤ 4 |

**Table S2. Sample sizes by provincial-level administrative divisions**

| **PLAD** | **Unweighted sample (n = 16,365), n (%)** | **Weighted sample (n = 15,000), n (%)** |
| --- | --- | --- |
| Anhui | 760 (4.6%) | 760 (5.1%) |
| Beijing | 240 (1.5%) | 84 (0.6%) |
| Chongqing | 402 (2.5%) | 386 (2.6%) |
| Fujian | 417 (2.5%) | 403 (2.7%) |
| Gansu | 338 (2.1%) | 295 (2.0%) |
| Guangdong | 1,115 (6.8%) | 1,115 (7.4%) |
| Guangxi | 734 (4.5%) | 734 (4.9%) |
| Guizhou | 591 (3.6%) | 591 (3.9%) |
| Hainan | 240 (1.5%) | 120 (0.8%) |
| Hebei | 903 (5.5%) | 903 (6.0%) |
| Heilongjiang | 347 (2.1%) | 296 (2.0%) |
| Henan | 1,351 (8.3%) | 1,351 (9.0%) |
| Hubei | 514 (3.1%) | 514 (3.4%) |
| Hunan | 781 (4.8%) | 781 (5.2%) |
| Inner Mongolia | 287 (1.8%) | 237 (1.6%) |
| Jiangsu | 690 (4.2%) | 690 (4.6%) |
| Jiangxi | 594 (3.6%) | 594 (4.0%) |
| Jilin | 293 (1.8%) | 222 (1.5%) |
| Liaoning | 383 (2.3%) | 358 (2.4%) |
| Ningxia | 240 (1.5%) | 94 (0.6%) |
| Qinghai | 240 (1.5%) | 86 (0.6%) |
| Shaanxi | 406 (2.5%) | 389 (2.6%) |
| Shandong | 992 (6.1%) | 992 (6.6%) |
| Shanghai | 240 (1.5%) | 107 (0.7%) |
| Shanxi | 406 (2.5%) | 396 (2.6%) |
| Sichuan | 907 (5.5%) | 907 (6.0%) |
| Tianjin | 240 (1.5%) | 99 (0.7%) |
| Xinjiang | 338 (2.1%) | 324 (2.2%) |
| Xizang | 240 (1.5%) | 38 (0.3%) |
| Yunnan | 588 (3.6%) | 588 (3.9%) |
| Zhejiang | 548 (3.3%) | 548 (3.7%) |
| PLAD, provincial-level administrative division; n (%), frequency with percentage. | | |

**Table S3. Sample characteristics by traditional cigarette and e-cigarette use status, unweighted**

| **Characteristic** | **Full sample (n_unweighted_ = 16,365), n (%)** | **Current dual users (n_unweighted_=1,207), n (%)** | **Current exclusive traditional cigarette users (n_unweighted_=2,913), n (%)** | **Current exclusive e-cigarette users (n_unweighted_=466), n (%)** | **Current nonusers (n_unweighted_=11,779), n (%)** |
| --- | --- | --- | --- | --- | --- |
| **Male, n (%)** | 8,176 (50.0%) | 999 (82.8%) | 2,563 (88.0%) | 333 (71.5%) | 4,281 (36.3%) |
| **Age, median (Q1, Q3)** | 17 (16, 17) | 18 (17, 18) | 17 (17, 18) | 17 (16, 18) | 17 (16, 18) |
| **Age (years), n (%)** |  |  |  |  |  |
| ≤ 16 | 2,174 (13.3%) | 77 (6.4%) | 246 (8.4%) | 56 (12.0%) | 1,795 (15.2%) |
| 16-17 | 4,054 (24.8%) | 247 (20.5%) | 693 (23.8%) | 127 (27.3%) | 2,987 (25.4%) |
| 17-18 | 5,897 (36.0%) | 436 (36.1%) | 1,278 (43.9%) | 158 (33.9%) | 4,025 (34.2%) |
| > 18 | 4,240 (25.9%) | 447 (37.0%) | 696 (23.9%) | 125 (26.8%) | 2,972 (25.2%) |
| **Residence in urban** | 9,421 (57.6%) | 768 (63.6%) | 1,987 (68.2%) | 226 (48.5%) | 6,440 (54.7%) |
| **School type, n (%)** |  |  |  |  |  |
| General high schools | 10,409 (63.6%) | 376 (31.2%) | 1,819 (62.4%) | 124 (26.6%) | 8,090 (68.7%) |
| Vocational high schools | 5,956 (36.4%) | 831 (68.8%) | 1,094 (37.6%) | 342 (73.4%) | 3,689 (31.3%) |
| **Grade, n (%)** |  |  |  |  |  |
| 10^th^ | 3,342 (20.4%) | 157 (13.0%) | 423 (14.5%) | 94 (20.2%) | 2,668 (22.7%) |
| 11^th^ | 5,043 (30.8%) | 328 (27.2%) | 1,029 (35.3%) | 158 (33.9%) | 3,528 (30.0%) |
| 12^th^ | 7,980 (48.8%) | 722 (59.8%) | 1,461 (50.2%) | 214 (45.9%) | 5,583 (47.4%) |
| n (%), frequency with percentage; IQR, interquartile range. | | | | | |

**Table S4. Prevalence of** **current traditional cigarette use among male and female high school students in China by provincial-level administrative division, weighted**

| **PLAD** | **Full sample, % (95% CI)** | **Male students, % (95% CI)** | **Female students, % (95% CI)** |
| --- | --- | --- | --- |
| Anhui | 24.2 (13.5, 39.5) | 39.8 (29.9, 50.7) | 7.4 (3.5, 14.9) |
| Beijing | 35.2 (12.6, 67.1) | 60.2 (52.2, 67.7) | 10.7 (1.4, 50.8) |
| Chongqing | 21.7 (11.2, 37.9) | 37.9 (31.3, 44.9) | 5.9 (3.4, 9.9) |
| Fujian | 23.5 (10.8, 44.0) | 41.8 (29.8, 55.0) | 5.1 (1.5, 16.3) |
| Gansu | 26.7 (12.6, 47.8) | 49.5 (40.9, 58.1) | 3.9 (0.7, 18.2) |
| Guangdong | 24.1 (10.6, 45.9) | 41.1 (28.7, 54.8) | 5.8 (1.8, 17.3) |
| Guangxi | 19.8 (9.4, 36.9) | 39.3 (31.9, 47.3) | 1.3 (0.3, 5.6) |
| Guizhou | 25.7 (12.8, 45.0) | 46.7 (38.9, 54.7) | 5.3 (1.4, 17.9) |
| Hainan | 29.9 (16.0, 48.7) | 50.7 (35.2, 66.0) | 8.3 (4.2, 15.7) |
| Hebei | 26.5 (13.7, 45.0) | 45.8 (33.4, 58.7) | 8.0 (4.0, 15.3) |
| Heilongjiang | 25.6 (12.1, 46.1) | 46.0 (39.8, 52.4) | 6.3 (1.8, 20.1) |
| Henan | 21.2 (10.8, 37.6) | 36.8 (26.5, 48.5) | 5.8 (1.9, 16.5) |
| Hubei | 22.2 (11.5, 38.4) | 39.0 (32.0, 46.5) | 4.4 (1.2, 15.1) |
| Hunan | 21.8 (11.1, 38.2) | 39.2 (32.0, 46.8) | 3.4 (1.2, 9.1) |
| Inner Mongolia | 21.1 (10.6, 37.6) | 34.9 (27.8, 42.8) | 7.8 (1.6, 30.2) |
| Jiangsu | 27.4 (12.6, 49.8) | 47.5 (34.1, 61.2) | 6.2 (2.3, 15.9) |
| Jiangxi | 23.6 (11.4, 42.4) | 42.1 (38.0, 46.2) | 1.8 (0.8, 4.1) |
| Jilin | 27.0 (14.0, 45.6) | 44.5 (33.8, 55.7) | 10.4 (4.9, 20.9) |
| Liaoning | 29.1 (12.2, 54.7) | 53.0 (39.1, 66.4) | 6.7 (1.9, 21.0) |
| Ningxia | 24.2 (12.6, 41.4) | 42.0 (37.4, 46.7) | 7.9 (2.8, 20.3) |
| Qinghai | 25.8 (14.4, 41.9) | 43.5 (38.4, 48.7) | 8.9 (2.9, 23.9) |
| Shaanxi | 28.0 (13.7, 48.8) | 48.2 (40.4, 56.2) | 7.5 (2.0, 24.4) |
| Shandong | 23.3 (11.2, 42.3) | 42.2 (30.8, 54.5) | 5.0 (2.5, 9.7) |
| Shanghai | 38.3 (18.0, 63.6) | 62.2 (52.2, 71.3) | 14.4 (4.4, 38.1) |
| Shanxi | 20.4 (7.8, 43.7) | 39.4 (21.8, 60.1) | 2.2 (0.5, 8.8) |
| Sichuan | 27.8 (15.8, 44.2) | 46.2 (37.8, 54.8) | 10.3 (4.6, 21.5) |
| Tianjin | 35.2 (15.8, 61.0) | 60.7 (51.1, 69.5) | 8.8 (4.7, 15.8) |
| Xinjiang | 28.9 (13.6, 51.2) | 51.5 (37.3, 65.5) | 9.7 (2.8, 28.6) |
| Xizang | 25.1 (11.4, 46.5) | 47.1 (42.4, 51.9) | 6.1 (1.5, 21.5) |
| Yunnan | 24.3 (12.5, 42.0) | 44.9 (42.0, 47.8) | 6.4 (2.2, 17.2) |
| Zhejiang | 26.3 (13.6, 44.6) | 43.5 (32.7, 54.9) | 8.8 (3.2, 21.9) |
| **Total** | **24.6 (23.9, 25.3)** | **43.1 (42.0, 44.2)** | **6.1 (5.6, 6.7)** |
| The presented results were derived from a weighted sample. PLAD, provincial-level administrative division; CI, confidence interval. | | | |

**Table S5. Prevalence of current e-cigarette use among male and female high school students in China by provincial-level administrative division, weighted**

| **PLAD** | **Full sample, % (95% CI)** | **Male students, % (95% CI)** | **Female students, % (95% CI)** |
| --- | --- | --- | --- |
| Anhui | 9.3 (4.5, 18.6) | 15.7 (8.4, 27.6) | 2.5 (0.7, 8.7) |
| Beijing | 13.4 (5.7, 28.5) | 20.2 (10.6, 35.0) | 6.8 (0.9, 36.9) |
| Chongqing | 5.8 (2.6, 12.4) | 9.7 (5.2, 17.4) | 2.1 (0.3, 12.0) |
| Fujian | 11.1 (5.5, 21.1) | 17.2 (10.3, 27.4) | 4.9 (0.9, 23.0) |
| Gansu | 9.0 (3.9, 19.3) | 14.9 (6.4, 31.1) | 3.0 (0.7, 12.5) |
| Guangdong | 7.7 (3.6, 15.8) | 12.4 (6.9, 21.5) | 2.6 (0.4, 13.7) |
| Guangxi | 7.4 (3.1, 16.5) | 12.1 (4.9, 26.7) | 2.9 (0.5, 14.5) |
| Guizhou | 8.3 (4.0, 16.4) | 13.7 (7.4, 24.1) | 3.0 (0.6, 13.7) |
| Hainan | 13.1 (6.7, 23.8) | 20.2 (10.8, 34.6) | 5.6 (1.5, 18.9) |
| Hebei | 11.2 (5.8, 20.5) | 18.6 (12.7, 26.3) | 4.1 (0.8, 19.3) |
| Heilongjiang | 8.6 (4.3, 16.5) | 14.7 (9.7, 21.8) | 2.8 (0.6, 12.9) |
| Henan | 9.7 (5.2, 17.4) | 14.6 (9.0, 22.8) | 4.9 (1.2, 17.6) |
| Hubei | 9.5 (4.6, 18.6) | 15.9 (8.4, 28.0) | 2.8 (0.5, 13.5) |
| Hunan | 8.2 (4.2, 15.3) | 13.2 (7.6, 22.1) | 2.9 (0.8, 10.4) |
| Inner Mongolia | 7.6 (3.5, 15.6) | 9.1 (4.7, 16.9) | 6.2 (1.3, 24.4) |
| Jiangsu | 12.6 (5.9, 24.9) | 20.6 (11.3, 34.6) | 4.2 (0.9, 17.5) |
| Jiangxi | 9.8 (5.3, 17.4) | 15.9 (12.8, 19.6) | 2.6 (0.5, 11.5) |
| Jilin | 8.1 (3.8, 16.3) | 12.9 (7.2, 21.9) | 3.6 (0.6, 19.2) |
| Liaoning | 13.2 (6.4, 25.1) | 22.4 (12.9, 36.1) | 4.5 (1.0, 18.5) |
| Ningxia | 9.5 (5.0, 17.4) | 14.1 (8.9, 21.7) | 5.2 (1.3, 18.7) |
| Qinghai | 10.5 (4.8, 21.6) | 14.0 (5.2, 32.6) | 7.1 (2.0, 22.0) |
| Shaanxi | 6.8 (3.5, 12.8) | 10.9 (6.6, 17.5) | 2.7 (0.6, 11.0) |
| Shandong | 9.8 (4.4, 20.4) | 17 .0 (8.1, 32.2) | 2.8 (0.7, 11.1) |
| Shanghai | 14.2 (5.3, 33.0) | 15.5 (4.5, 41.8) | 12.8 (2.5, 46.1) |
| Shanxi | 9.3 (3.6, 22.0) | 18.1 (8.2, 35.3) | 0.8 (0.1, 6.1) |
| Sichuan | 10.3 (4.7, 21) | 15.8 (6.8, 32.8) | 4.9 (1.2, 17.9) |
| Tianjin | 12.7 (6.2, 24.3) | 20.8 (12.4, 32.8) | 4.3 (0.8, 19.7) |
| Xinjiang | 10.9 (5.1, 21.8) | 16.8 (8.3, 31.2) | 5.9 (1.4, 21.4) |
| Xizang | 10.3 (4.9, 20.4) | 17.5 (11.9, 24.9) | 4.0 (0.9, 16.5) |
| Yunnan | 8.8 (4.5, 16.6) | 16.4 (11.3, 23.3) | 2.2 (0.6, 7.8) |
| Zhejiang | 10.6 (5.1, 20.6) | 18.5 (11.3, 28.7) | 2.6 (0.5, 12.9) |
| **Total** | **9.5 (9.1, 10.0)** | **15.5 (14.7, 16.4)** | **3.5 (3.1, 4.0)** |
| The presented results were derived from a weighted sample. PLAD, provincial-level administrative division; CI, confidence interval. | | | |

## Table S6. Prevalence of current dual use among male and female high school students in China by provincial-level administrative division, weighted

| **PLAD** | **Full sample, % (95% CI)** | **Male students, % (95% CI)** | **Female students, % (95% CI)** |
| --- | --- | --- | --- |
| Anhui | 6.6 (3.3, 12.7) | 11.2 (6.7, 18.0) | 1.6 (0.4, 5.8) |
| Beijing | 11.1 (4.9, 23.4) | 17.1 (10.9, 25.6) | 5.3 (0.7, 30.2) |
| Chongqing | 4.1 (1.8, 9.0) | 7.2 (4.2, 12.0) | 1.0 (0.2, 6.2) |
| Fujian | 7.6 (3.3, 16.4) | 12.1 (5.8, 23.5) | 3.0 (0.5, 16.8) |
| Gansu | 6.8 (2.9, 15.0) | 11.9 (5.1, 25.1) | 1.7 (0.4, 7.3) |
| Guangdong | 5.2 (2.3, 11.5) | 9.0 (4.9, 15.9) | 1.1 (0.2, 6.9) |
| Guangxi | 4.2 (1.5, 11.2) | 8.4 (3.5, 19.1) | 0.3 (0.0, 1.7) |
| Guizhou | 7.3 (3.5, 14.7) | 12.0 (6.4, 21.4) | 2.7 (0.5, 14.0) |
| Hainan | 9.9 (4.8, 19.3) | 15.5 (7.3, 29.8) | 4.0 (1.2, 12.8) |
| Hebei | 7.8 (3.9, 15.0) | 13.4 (8.5, 20.4) | 2.4 (0.5, 10.4) |
| Heilongjiang | 7.0 (3.6, 13.4) | 11.9 (8.9, 15.7) | 2.5 (0.5, 12.4) |
| Henan | 6.4 (3.3, 12.2) | 10.3 (5.9, 17.2) | 2.7 (0.6, 10.7) |
| Hubei | 7.0 (3.3, 14.2) | 12.5 (6.8, 22.0) | 1.2 (0.2, 7.6) |
| Hunan | 5.6 (2.7, 11.2) | 9.7 (5.4, 17.0) | 1.3 (0.3, 5.7) |
| Inner Mongolia | 5.3 (2.3, 11.7) | 6.4 (2.6, 14.7) | 4.2 (0.9, 17.8) |
| Jiangsu | 9.1 (4.0, 19.7) | 15.3 (7.6, 28.3) | 2.7 (0.6, 10.5) |
| Jiangxi | 6.9 (3.3, 13.9) | 12.5 (8.8, 17.3) | 0.4 (0.1, 2.6) |
| Jilin | 4.9 (2.3, 10.2) | 8.2 (4.8, 13.6) | 1.9 (0.4, 9.1) |
| Liaoning | 9.6 (4.3, 20.2) | 17.5 (9.7, 29.6) | 2.3 (0.5, 9.7) |
| Ningxia | 8.4 (4.6, 15.0) | 12.4 (9.1, 16.7) | 4.8 (1.2, 17.2) |
| Qinghai | 8.3 (3.6, 17.8) | 11.6 (4.2, 27.9) | 5.1 (1.4, 16.5) |
| Shaanxi | 4.9 (2.4, 9.7) | 8.0 (4.2, 14.5) | 1.8 (0.4, 7.4) |
| Shandong | 7.3 (2.9, 17.2) | 13.3 (5.6, 28.7) | 1.4 (0.3, 6.0) |
| Shanghai | 11.7 (4.4, 27.8) | 15.2 (4.3, 41.5) | 8.2 (1.7, 30.8) |
| Shanxi | 5.8 (1.7, 17.6) | 11.4 (3.9, 29.2) | 0.4 (0.1, 3.1) |
| Sichuan | 8.0 (3.7, 16.8) | 12.9 (5.6, 26.8) | 3.4 (0.9, 12.6) |
| Tianjin | 9.3 (4.6, 18.1) | 15.6 (11.7, 20.6) | 2.8 (0.4, 15.8) |
| Xinjiang | 9.6 (4.5, 19.1) | 15.2 (8.0, 27.0) | 4.8 (1.1, 18.2) |
| Xizang | 8.9 (4.1, 18.2) | 15.6 (11.5, 20.9) | 3.1 (0.6, 15) |
| Yunnan | 6.5 (3.0, 13.2) | 12.8 (7.9, 19.9) | 1.0 (0.2, 3.7) |
| Zhejiang | 8.0 (4.0, 15.4) | 13.4 (8.5, 20.4) | 2.6 (0.5, 12.9) |
| **Total** | **6.9 (6.5, 7.3)** | **11.7 (11.0, 12.4)** | **2.1 (1.8, 2.4)** |
| The presented results were derived from a weighted sample. PLAD, provincial-level administrative division; CI, confidence interval. | | | |

**Table S7. Factors potentially associated with** **current dual use, exclusive traditional cigarette use and exclusive e-cigarette use among high school students in China, weighted**

|  | **Full sample (n_weighted_ = 15,000), n (%)** | **Current dual users (n_weighted_=1,029), n (%)** | **Current exclusive traditional cigarette users**  **(n_weighted_=2,655), n (%)** | **Current exclusive e-cigarette users (n_weighted_=398), n (%)** | **Current nonusers (n_weighted_=10,918), n (%)** |
| --- | --- | --- | --- | --- | --- |
| **Behavioral factors** | | | | | |
| **Physical activity** |  |  |  |  |  |
| Above average | 5,392 (35.9%) | 459 (44.3%) | 1,228 (46.3%) | 149 (37.5%) | 3,559 (32.6%) |
| Below average | 9,608 (64.1%) | 573 (55.7%) | 1,427 (53.7%) | 249 (62.5%) | 7,359 (67.4%) |
| **Screen time** |  |  |  |  |  |
| Appropriate | 6,648 (44.3%) | 549 (53.3%) | 1,316 (49.6%) | 214 (53.9%) | 4,569 (41.8%) |
| Excessive | 8,352 (55.7%) | 480 (46.7%) | 1,339 (50.4%) | 184 (46.1%) | 6,349 (58.2%) |
| **Sleep duration** |  |  |  |  |  |
| Sufficient sleep | 4,893 (32.6%) | 262 (25.5%) | 699 (26.3%) | 122 (30.7%) | 3,810 (34.9%) |
| Insufficient sleep | 10,107 (67.4%) | 767 (74.5%) | 1,956 (73.7%) | 276 (69.3%) | 7,108 (65.1%) |
| **Diet** |  |  |  |  |  |
| Healthy | 7,557 (50.4%) | 430 (41.8%) | 1,213 (45.7%) | 165 (41.5%) | 5,749 (52.7%) |
| Unhealthy | 7,443 (49.6%) | 599 (58.2%) | 1,442 (54.3%) | 233 (58.5%) | 5,169 (47.3%) |
| **Alcohol use** |  |  |  |  |  |
| Never use | 4,567 (30.4%) | 57 (5.6%) | 236 (8.9%) | 40 (10.2%) | 4,232 (38.8%) |
| Occasionally use | 9,122 (60.8%) | 741 (72.0%) | 1,977 (74.5%) | 277 (69.5%) | 6,128 (56.1%) |
| Habitually use | 1,311 (8.7%) | 231 (22.5%) | 442 (16.6%) | 81 (20.3%) | 558 (5.1%) |
| **Fight** |  |  |  |  |  |
| Never | 13,355 (89%) | 789 (76.6%) | 2,143 (80.7%) | 312 (78.3%) | 10,112 (92.6%) |
| Occasionally | 1,408 (9.4%) | 191 (18.5%) | 438 (16.5%) | 72 (18.2%) | 707 (6.5%) |
| Habitual | 237 (1.6%) | 50 (4.8%) | 74 (2.8%) | 14 (3.5%) | 99 (0.9%) |
| **Psychological factors** | | | | | |
| **Social self-control** |  |  |  |  |  |
| Strong | 3,440 (22.9%) | 163 (15.8%) | 518 (19.5%) | 82 (20.6%) | 2,678 (24.5%) |
| Moderate | 7,679 (51.2%) | 498 (48.3%) | 1338 (50.4%) | 157 (39.4%) | 5,687 (52.1%) |
| Weak | 3,881 (25.9%) | 369 (35.8%) | 799 (30.1%) | 159 (40%) | 2,554 (23.4%) |
| **Self-esteem** |  |  |  |  |  |
| Strong | 4,179 (27.9%) | 442 (42.9%) | 1,056 (39.8%) | 125 (31.4%) | 2,556 (23.4%) |
| Moderate | 7,691 (51.3%) | 430 (41.8%) | 1,142 (43.0%) | 190 (47.8%) | 5,930 (54.3%) |
| Weak | 3,130 (20.9%) | 157 (15.3%) | 457 (17.2%) | 83 (20.8%) | 2,433 (22.3%) |
| **Self-efficacy** |  |  |  |  |  |
| Strong | 3,772 (25.1%) | 435 (42.2%) | 961 (36.2%) | 138 (34.6%) | 2,239 (20.5%) |
| Moderate | 8,067 (53.8%) | 489 (47.5%) | 1,366 (51.4%) | 193 (48.4%) | 6,019 (55.1%) |
| Weak | 3,162 (21.1%) | 105 (10.2%) | 328 (12.4%) | 67 (17.0%) | 2,661 (24.4%) |
| **Stress** |  |  |  |  |  |
| Strong | 2,987 (19.9%) | 153 (14.9%) | 415 (15.6%) | 88 (22.1%) | 2,331 (21.3%) |
| Moderate | 7,835 (52.2%) | 555 (54.0%) | 1,342 (50.5%) | 213 (53.5%) | 5,725 (52.4%) |
| Weak | 4,178 (27.9%) | 321 (31.2%) | 898 (33.8%) | 97 (24.4%) | 2,862 (26.2%) |
| **Depression** |  |  |  |  |  |
| Severe | 396 (2.6%) | 19 (1.8%) | 71 (2.7%) | 15 (3.8%) | 291 (2.7%) |
| Moderately severe | 1347 (9.0%) | 118 (11.4%) | 227 (8.6%) | 50 (12.7%) | 952 (8.7%) |
| Moderate | 3,313 (22.1%) | 239 (23.2%) | 561 (21.1%) | 93 (23.5%) | 2,420 (22.2%) |
| Mild | 6,051 (40.3%) | 361 (35.1%) | 1,005 (37.8%) | 143 (35.9%) | 4,543 (41.6%) |
| Minimal | 3,893 (26.0%) | 293 (28.5%) | 791 (29.8%) | 96 (24.1%) | 2,712 (24.8%) |
| The presented results were derived from a weighted sample. n (%), frequency with percentage. | | | | | |

**Table S8. Factors associated with current dual use, exclusive traditional cigarette use and exclusive e-cigarette use among high school students in China, weighted**

| **Factor** | **Current dual use** | | **Current exclusive traditional cigarette use** | | **Current exclusive e-cigarette use** | |
| --- | --- | --- | --- | --- | --- | --- |
|  | **OR (95% CI)** | **P value** | **OR (95% CI)** | **P value** | **OR (95% CI)** | **P value** |
| **Behavioral factors** | | | | | | |
| **Physical activity (ref: above average)** |  |  |  |  |  |  |
| Below average | 0.99 (0.85, 1.16) | 0.888 | 0.96 (0.85, 1.09) | 0.559 | 1.12 (0.91, 1.38) | 0.298 |
| **Screen time (ref: appropriate)** |  |  |  |  |  |  |
| Excessive | 0.70 (0.61, 0.80) | < 0.001 | 0.81 (0.73, 0.89) | < 0.001 | 0.62 (0.48, 0.79) | < 0.001 |
| **Sleep duration (ref: sufficient)** |  |  |  |  |  |  |
| Insufficient | 1.18 (1.00, 1.38) | 0.050 | 1.17 (1.05, 1.30) | 0.005 | 0.93 (0.75, 1.16) | 0.520 |
| **Diet (ref: healthy)** |  |  |  |  |  |  |
| Unhealthy | 1.34 (1.15, 1.56) | < 0.001 | 1.25 (1.12, 1.40) | < 0.001 | 1.42 (1.15, 1.76) | 0.001 |
| **Alcohol use (ref: never use)** |  |  |  |  |  |  |
| Occasionally use | 5.53 (4.27, 7.17) | < 0.001 | 3.71 (3.15, 4.37) | < 0.001 | 3.50 (2.54, 4.82) | < 0.001 |
| Habitually use | 12.71 (9.24, 17.47) | < 0.001 | 6.75 (5.23, 8.72) | < 0.001 | 7.77 (5.16, 11.69) | < 0.001 |
| **Fight (ref: never)** |  |  |  |  |  |  |
| Occasional | 1.69 (1.41, 2.03) | < 0.001 | 1.58 (1.35, 1.86) | < 0.001 | 1.63 (1.21, 2.19) | 0.002 |
| Habitual | 1.83 (1.18, 2.82) | 0.007 | 1.40 (0.88, 2.23) | 0.152 | 1.25 (0.61, 2.55) | 0.538 |
| **Psychological factors** | | | | | | |
| **Social self-control (ref: moderate)** |  |  |  |  |  |  |
| Strong | 0.69 (0.57, 0.82) | < 0.001 | 0.77 (0.68, 0.87) | < 0.001 | 1.14 (0.90, 1.46) | 0.270 |
| Weak | 1.13 (0.96, 1.33) | 0.132 | 1.02 (0.91, 1.14) | 0.727 | 1.61 (1.25, 2.08) | < 0.001 |
| **Self-esteem (ref: moderate)** |  |  |  |  |  |  |
| Strong | 1.62 (1.36, 1.94) | < 0.001 | 1.51 (1.33, 1.70) | < 0.001 | 1.07 (0.82, 1.39) | 0.627 |
| Weak | 1.10 (0.90, 1.34) | 0.360 | 1.15 (0.98, 1.34) | 0.086 | 1.06 (0.78, 1.43) | 0.705 |
| **Self-efficacy (ref: moderate)** |  |  |  |  |  |  |
| Strong | 1.61 (1.38, 1.88) | < 0.001 | 1.36 (1.19, 1.56) | < 0.001 | 1.53 (1.21, 1.94) | < 0.001 |
| Weak | 0.78 (0.62, 0.97) | 0.027 | 0.82 (0.70, 0.97) | 0.019 | 0.97 (0.69, 1.36) | 0.859 |
| **Stress (ref: moderate)** |  |  |  |  |  |  |
| Strong | 0.78 (0.64, 0.95) | 0.014 | 0.92 (0.79, 1.07) | 0.283 | 1.05 (0.77, 1.41) | 0.769 |
| Weak | 1.07 (0.89, 1.28) | 0.467 | 1.17 (1.03, 1.34) | 0.017 | 0.89 (0.69, 1.15) | 0.380 |
| **Depression (ref: minimal)** |  |  |  |  |  |  |
| Severe | 0.61 (0.33, 1.10) | 0.100 | 1.08 (0.74, 1.58) | 0.701 | 1.04 (0.59, 1.84) | 0.880 |
| Moderately severe | 1.21 (0.90, 1.61) | 0.199 | 1.02 (0.81, 1.29) | 0.848 | 1.04 (0.68, 1.60) | 0.864 |
| Moderate | 0.98 (0.79, 1.22) | 0.890 | 0.94 (0.79, 1.12) | 0.492 | 0.86 (0.60, 1.25) | 0.434 |
| Mild | 0.88 (0.72, 1.09) | 0.237 | 0.96 (0.84, 1.10) | 0.558 | 0.87 (0.62, 1.23) | 0.428 |
| The presented results were derived from a weighted mutually adjusted multinomial logistic regression model with non-use of either e-cigarettes or traditional cigarettes as the reference category. The model controlled for age, sex, current parental tobacco use, BMI and its quadratic term, and fixed effects for PLADs. To mitigate multicollinearity, we performed centering on BMI by subtracting each individual's BMI from the mean BMI of all participants and then included the centered BMI and its quadratic term in the model. [15, 16] The variance inflation factors for exposure variables ranged from 1.04 to 1.99, indicating no significant multicollinearity.[17] OR, odds ratio; CI, confidence interval; ref, reference; BMI: body mass index; PLAD, provincial-level administrative division. | | | | | | |

**Table S9. Sex subgroup** **analysis of factors associated with current dual use, exclusive traditional cigarette use and exclusive e-cigarette use among high school students in China, weighted**

| **Factors** | **Current dual use** | | | | **Current exclusive traditional cigarette use** | | | | **Current exclusive e-cigarette use** | | | |
| --- | --- | --- | --- | --- | --- | --- | --- | --- | --- | --- | --- | --- |
|  | **Male** | | **Female** | | **Male** | | **Female** | | **Male** | | **Female** | |
|  | **OR (95% CI)** | ***P* value** | **OR (95% CI)** | ***P* value** | **OR (95% CI)** | ***P* value** | **OR (95% CI)** | ***P* value** | **OR (95% CI)** | ***P* value** | **OR (95% CI)** | ***P* value** |
| **Behavioral factors** | | | | | | | | | | | | |
| **Physical activity (ref: above average)** |  |  |  |  |  |  |  |  |  |  |  |  |
| Below average | 1.00 (0.84, 1.18) | 0.972 | 1.00 (0.71, 1.41) | 0.986 | 0.99 (0.86, 1.13) | 0.829 | 0.89 (0.61, 1.29) | 0.528 | 1.13 (0.89, 1.43) | 0.302 | 1.07 (0.65, 1.77) | 0.777 |
| **Screen time (ref: appropriate)** |  |  |  |  |  |  |  |  |  |  |  |  |
| Excessive | 0.69 (0.59, 0.80) | <0.001 | 0.78 (0.57, 1.07) | 0.117 | 0.80 (0.71, 0.89) | <0.001 | 0.83 (0.63, 1.08) | 0.167 | 0.67 (0.49, 0.91) | 0.011 | 0.51 (0.35, 0.74) | 0.001 |
| **Sleep duration (ref: sufficient)** |  |  |  |  |  |  |  |  |  |  |  |  |
| Insufficient | 1.15 (0.95, 1.38) | 0.141 | 1.21 (0.87, 1.70) | 0.256 | 1.11 (0.98, 1.26) | 0.102 | 1.40 (1.10, 1.78) | 0.007 | 0.81(0.62, 1.07) | 0.133 | 1.20 (0.84, 1.71) | 0.312 |
| **Diet (ref: healthy)** |  |  |  |  |  |  |  |  |  |  |  |  |
| Unhealthy | 1.38 (1.16, 1.64) | <0.001 | 1.13 (0.81, 1.58) | 0.467 | 1.26 (1.11, 1.43) | <0.001 | 1.26 (0.98, 1.62) | 0.076 | 1.47 (1.14, 1.88) | 0.003 | 1.39 (0.93, 2.06) | 0.107 |
| **Alcohol use (ref: never use)** |  |  |  |  |  |  |  |  |  |  |  |  |
| Occasionally use | 4.72 (3.55, 6.28) | <0.001 | 11.33 (5.45, 23.53) | <0.001 | 3.45 (2.87, 4.16) | <0.001 | 5.46 (3.76, 7.93) | <0.001 | 3.89 (2.51, 6.02) | <0.001 | 2.80 (1.77, 4.44) | <0.001 |
| Habitually use | 11.17 (7.77, 16.05) | <0.001 | 24.13 (10.89, 53.48) | <0.001 | 6.40 (4.72, 8.67) | <0.001 | 10.32 (6.31, 16.90) | <0.001 | 8.30 (4.76, 14.46) | <0.001 | 6.98 (4.03, 12.10) | <0.001 |
| **Fight (ref: never)** |  |  |  |  |  |  |  |  |  |  |  |  |
| Occasional | 1.68 (1.36, 2.08) | <0.001 | 1.53 (0.97, 2.41) | 0.065 | 1.52 (1.27, 1.81) | <0.001 | 1.98 (1.37, 2.87) | <0.001 | 1.66 (1.20, 2.28) | 0.002 | 1.54 (0.76, 3.09) | 0.226 |
| Habitual | 1.82 (1.08, 3.06) | 0.024 | 1.50 (0.72, 3.13) | 0.278 | 1.20 (0.69, 2.09) | 0.513 | 2.27 (1.08, 4.76) | 0.031 | 1.20 (0.53, 2.72) | 0.664 | 1.29 (0.33, 4.94) | 0.713 |
| **Psychological factors** | | | | | | | | | | | | |
| **Social self-control (ref: moderate)** |  |  |  |  |  |  |  |  |  |  |  |  |
| Strong | 0.70 (0.57, 0.85) | <0.001 | 0.67 (0.38, 1.18) | 0.162 | 0.79 (0.69, 0.91) | 0.001 | 0.62 (0.43, 0.91) | 0.015 | 1.08 (0.82, 1.42) | 0.567 | 1.35 (0.76, 2.41) | 0.308 |
| Weak | 1.18 (0.99, 1.42) | 0.067 | 0.95 (0.64, 1.39) | 0.786 | 1.03 (0.90, 1.18) | 0.644 | 1.01 (0.77, 1.34) | 0.918 | 1.60 (1.20, 2.15) | 0.002 | 1.59 (0.95, 2.66) | 0.080 |
| **Self-esteem (ref: moderate)** |  |  |  |  |  |  |  |  |  |  |  |  |
| Strong | 1.67 (1.36, 2.06) | <0.001 | 1.37 (1.00, 1.90) | 0.053 | 1.46 (1.27, 1.67) | <0.001 | 1.73 (1.36, 2.21) | <0.001 | 1.00 (0.72, 1.40) | 0.988 | 1.21 (0.78, 1.88) | 0.387 |
| Weak | 1.11 (0.89, 1.38) | 0.355 | 0.93 (0.60, 1.43) | 0.736 | 1.09 (0.92, 1.29) | 0.334 | 1.52 (1.01, 2.29) | 0.043 | 1.02 (0.71, 1.47) | 0.897 | 1.08 (0.64, 1.81) | 0.778 |
| **Self-efficacy (ref: moderate)** |  |  |  |  |  |  |  |  |  |  |  |  |
| Strong | 1.52 (1.28, 1.81) | <0.001 | 1.93 (1.42, 2.62) | <0.001 | 1.31 (1.12, 1.53) | 0.001 | 1.48 (1.13, 1.93) | 0.005 | 1.45 (1.08, 1.94) | 0.013 | 1.77 (1.16, 2.71) | 0.009 |
| Weak | 0.90 (0.71, 1.15) | 0.399 | 0.46 (0.27, 0.78) | 0.004 | 0.87 (0.72, 1.05) | 0.140 | 0.73 (0.52, 1.02) | 0.062 | 0.97 (0.63, 1.48) | 0.880 | 0.99 (0.57, 1.73) | 0.974 |
| **Stress (ref: moderate)** |  |  |  |  |  |  |  |  |  |  |  |  |
| Strong | 0.68 (0.54, 0.85) | 0.001 | 1.12 (0.74, 1.69) | 0.599 | 0.84 (0.71, 1.00) | 0.050 | 1.11 (0.82, 1.50) | 0.494 | 0.83 (0.56, 1.23) | 0.352 | 1.52 (0.96, 2.41) | 0.071 |
| Weak | 1.12 (0.92, 1.36) | 0.262 | 0.77 (0.47, 1.25) | 0.282 | 1.16 (1.00, 1.35) | 0.054 | 1.32 (1.01, 1.73) | 0.043 | 0.91 (0.67, 1.23) | 0.530 | 0.81 (0.50, 1.31) | 0.385 |
| **Depression (ref: minimal)** |  |  |  |  |  |  |  |  |  |  |  |  |
| Severe | 0.61 (0.30, 1.24) | 0.168 | 0.52 (0.19, 1.43) | 0.201 | 1.05 (0.68, 1.63) | 0.830 | 1.15 (0.56, 2.34) | 0.704 | 1.17 (0.60, 2.29) | 0.647 | 0.81 (0.30, 2.23) | 0.683 |
| Moderately severe | 1.20 (0.87, 1.65) | 0.268 | 1.16 (0.62, 2.15) | 0.642 | 1.03 (0.79, 1.35) | 0.814 | 0.85 (0.53, 1.35) | 0.492 | 0.94 (0.56, 1.58) | 0.804 | 1.19 (0.51, 2.74) | 0.684 |
| Moderate | 1.02 (0.80, 1.30) | 0.899 | 0.90 (0.56, 1.45) | 0.674 | 0.99 (0.81, 1.22) | 0.961 | 0.74 (0.50, 1.09) | 0.124 | 0.88 (0.55, 1.40) | 0.586 | 0.89 (0.47, 1.69) | 0.719 |
| Mild | 0.91 (0.71, 1.16) | 0.431 | 0.77 (0.53, 1.11) | 0.153 | 0.98 (0.85, 1.13) | 0.787 | 0.92 (0.65, 1.30) | 0.621 | 0.89 (0.60, 1.33) | 0.573 | 0.83 (0.41, 1.69) | 0.607 |
| The presented results were derived from a weighted mutually adjusted multinomial logistic regression model with non-use of either e-cigarettes or traditional cigarettes as the reference category. The model controlled for age, BMI and its quadratic term, current parental tobacco use, and fixed effects for PLADs. The variance inflation factors for exposure variables ranged from 1.02 to 1.88 among males and 1.01 to 2.16 among females, indicating no significant multicollinearity.[17] OR, odds ratio; CI, confidence interval; ref, reference; PLAD, provincial-level administrative division. | | | | | | | | | | | | |

**Table S10. Age subgroup analysis of factors associated with** **current dual use, exclusive traditional cigarette use and exclusive e-cigarette use among high school students in China: weighted**

| **Factors** | **Current dual use** | | | | **Current exclusive traditional cigarette use** | | | | **Current exclusive e-cigarette use** | | | |
| --- | --- | --- | --- | --- | --- | --- | --- | --- | --- | --- | --- | --- |
|  | **Below mean age (17.3 years)** | | **Above mean age (17.3 years)** | | **Below mean age (17.3 years)** | | **Above mean age (17.3 years)** | | **Below mean age (17.3 years)** | | **Above mean age (17.3 years)** | |
|  | **OR (95% CI)** | ***P* value** | **OR (95% CI)** | ***P* value** | **OR (95% CI)** | ***P* value** | **OR (95% CI)** | ***P* value** | **OR (95% CI)** | ***P* value** | **OR (95% CI)** | ***P* value** |
| **Behavioral factors** | | | | | | | | | | | | |
| **Physical activity (ref: above average)** |  |  |  |  |  |  |  |  |  |  |  |  |
| Below average | 0.79 (0.58, 1.08) | 0.137 | 1.10 (0.92, 1.31) | 0.306 | 1.05 (0.86, 1.28) | 0.614 | 0.90 (0.77, 1.04) | 0.142 | 1.16 (0.82, 1.63) | 0.411 | 1.09 (0.82, 1.44) | 0.560 |
| **Screen time (ref: appropriate)** |  |  |  |  |  |  |  |  |  |  |  |  |
| Excessive | 0.69 (0.55, 0.85) | 0.001 | 0.71 (0.59, 0.86) | 0.001 | 0.77 (0.66, 0.88) | <0.001 | 0.84 (0.71, 0.99) | 0.034 | 0.60 (0.44, 0.82) | 0.001 | 0.66 (0.45, 0.96) | 0.032 |
| **Sleep duration (ref: sufficient)** |  |  |  |  |  |  |  |  |  |  |  |  |
| Insufficient | 1.35 (1.06, 1.73) | 0.016 | 1.06 (0.86, 1.32) | 0.582 | 1.24 (1.03, 1.49) | 0.024 | 1.11 (0.96, 1.29) | 0.142 | 0.73 (0.54, 0.97) | 0.033 | 1.17 (0.84, 1.63) | 0.355 |
| **Diet (ref: healthy)** |  |  |  |  |  |  |  |  |  |  |  |  |
| Unhealthy | 1.23 (0.98, 1.54) | 0.080 | 1.44 (1.18, 1.76) | <0.001 | 1.17 (1.00, 1.37) | 0.054 | 1.34 (1.15, 1.57) | <0.001 | 1.35 (1.05, 1.74) | 0.019 | 1.50 (1.06, 2.13) | 0.022 |
| **Alcohol use (ref: never use)** |  |  |  |  |  |  |  |  |  |  |  |  |
| Occasionally use | 7.91 (4.69, 13.33) | <0.001 | 4.48 (3.17, 6.33) | <0.001 | 3.68 (2.95, 4.60) | <0.001 | 3.88 (3.01, 5.01) | <0.001 | 3.53 (2.22, 5.62) | <0.001 | 3.48 (2.18, 5.58) | <0.001 |
| Habitually use | 16.38 (8.53, 31.44) | <0.001 | 10.93 (7.19, 16.64) | <0.001 | 6.09 (4.20, 8.82) | <0.001 | 7.68 (5.49, 10.75) | <0.001 | 6.42 (3.60, 11.45) | <0.001 | 9.23 (4.85, 17.57) | <0.001 |
| **Fight (ref: never)** |  |  |  |  |  |  |  |  |  |  |  |  |
| Occasional | 2.01 (1.45, 2.77) | <0.001 | 1.50 (1.13, 1.99) | 0.005 | 1.53 (1.20, 1.97) | 0.001 | 1.66 (1.32, 2.08) | <0.001 | 1.34 (0.89, 2.02) | 0.154 | 1.95 (1.28, 2.99) | 0.002 |
| Habitual | 2.40 (1.08, 5.32) | 0.032 | 1.59 (0.88, 2.89) | 0.126 | 1.68 (0.83, 3.39) | 0.146 | 1.20 (0.68, 2.09) | 0.525 | 1.61 (0.61, 4.27) | 0.335 | 1.10 (0.46, 2.66) | 0.826 |
| **Psychological factors** | | | | | | | | | | | | |
| **Social self-control (ref: moderate)** |  |  |  |  |  |  |  |  |  |  |  |  |
| Strong | 0.69 (0.50, 0.95) | 0.024 | 0.68 (0.55, 0.86) | 0.001 | 0.65 (0.53, 0.78) | <0.001 | 0.87 (0.74, 1.02) | 0.079 | 1.57 (1.10, 2.23) | 0.013 | 0.82 (0.55, 1.22) | 0.324 |
| Weak | 0.97 (0.77, 1.24) | 0.824 | 1.25 (1.00, 1.56) | 0.047 | 0.89 (0.76, 1.04) | 0.151 | 1.12 (0.94, 1.33) | 0.201 | 1.72 (1.22, 2.42) | 0.002 | 1.51 (1.10, 2.07) | 0.012 |
| **Self-esteem (ref: moderate)** |  |  |  |  |  |  |  |  |  |  |  |  |
| Strong | 1.83 (1.44, 2.32) | <0.001 | 1.48 (1.16, 1.90) | 0.002 | 1.67 (1.37, 2.04) | <0.001 | 1.37 (1.18, 1.59) | <0.001 | 1.13 (0.80, 1.60) | 0.475 | 1.04 (0.69, 1.57) | 0.835 |
| Weak | 1.08 (0.76, 1.54) | 0.664 | 1.13 (0.88, 1.44) | 0.326 | 1.03 (0.81, 1.31) | 0.789 | 1.26 (1.03, 1.53) | 0.024 | 1.11 (0.72, 1.72) | 0.630 | 1.01 (0.63, 1.61) | 0.971 |
| **Self-efficacy (ref: moderate)** |  |  |  |  |  |  |  |  |  |  |  |  |
| Strong | 1.51 (1.16, 1.97) | 0.002 | 1.67 (1.34, 2.08) | <0.001 | 1.23 (1.02, 1.48) | 0.031 | 1.50 (1.24, 1.81) | <0.001 | 1.60 (1.12, 2.28) | 0.010 | 1.47 (1.03, 2.09) | 0.032 |
| Weak | 0.88 (0.63, 1.23) | 0.465 | 0.71 (0.54, 0.94) | 0.016 | 1.00 (0.81, 1.24) | 0.989 | 0.69 (0.55, 0.86) | 0.001 | 0.97 (0.63, 1.48) | 0.870 | 1.00 (0.62, 1.63) | 0.988 |
| **Stress (ref: moderate)** |  |  |  |  |  |  |  |  |  |  |  |  |
| Strong | 0.95 (0.68, 1.32) | 0.764 | 0.70 (0.55, 0.90) | 0.007 | 0.88 (0.70, 1.09) | 0.237 | 0.94 (0.75, 1.17) | 0570 | 1.15 (0.77, 1.72) | 0.495 | 0.96 (0.66, 1.39) | 0.819 |
| Weak | 1.14 (0.88, 1.48) | 0.329 | 1.01 (0.81, 1.25) | 0.955 | 1.24 (1.04, 1.47) | 0.018 | 1.15 (0.97, 1.36) | 0.118 | 0.75 (0.53, 1.07) | 0.116 | 1.00 (0.72, 1.40) | 0.976 |
| **Depression (ref: minimal)** |  |  |  |  |  |  |  |  |  |  |  |  |
| Severe | 0.24 (0.08, 0.71) | 0.010 | 0.97 (0.48, 1.96) | 0.925 | 0.72 (0.42, 1.21) | 0.210 | 1.45 (0.91, 2.30) | 0.114 | 0.55 (0.22, 1.36) | 0.193 | 1.79 (0.82, 3.90) | 0.143 |
| Moderately severe | 0.94 (0.60, 1.45) | 0.765 | 1.39 (1.00, 1.93) | 0.047 | 0.89 (0.64, 1.24) | 0.496 | 1.14 (0.87, 1.50) | 0.351 | 0.83 (0.47, 1.47) | 0.526 | 1.25 (0.70, 2.21) | 0.445 |
| Moderate | 0.85 (0.60, 1.19) | 0.339 | 1.12 (0.84, 1.51) | 0.437 | 0.76 (0.60, 0.98) | 0.032 | 1.11 (0.86, 1.43) | 0.425 | 0.78 (0.47, 1.30) | 0.331 | 0.95 (0.60, 1.52) | 0.838 |
| Mild | 0.72 (0.52, 0.99) | 0.045 | 0.99 (0.76, 1.29) | 0.968 | 0.89 (0.72, 1.08) | 0.237 | 1.01 (0.86, 1.19) | 0.877 | 0.84 (0.54, 1.31) | 0.441 | 0.89 (0.58, 1.35) | 0.576 |
| The presented results were derived from a weighted mutually adjusted multinomial logistic regression model with non-use of either e-cigarettes or traditional cigarettes as the reference category. The model controlled for sex, BMI and its quadratic term, current parental tobacco use, and fixed effects for PLADs. The variance inflation factors for exposure variables ranged from 1.04 to 2.01 for below average and 1.04 to 1.98 for above average, indicating no significant multicollinearity.[17] OR, odds ratio; CI, confidence interval; ref, reference; PLAD, provincial-level administrative division. | | | | | | | | | | | | |

**SUPPLEMENTARY REFERENCES**

1.	**Homepage for KuRunData** [<https://www.kurundata.com/en/index.html>]

2.	Yu F, Geldsetzer P, Meierkord A, Yang J, Chen Q, Jiao L, Abou-Arraj NE, Pan A, Wang C, Bärnighausen T *et al*: **Knowledge About COVID-19 Among Adults in China: Cross-sectional Online Survey**. *J Med Internet Res* 2021, **23**(4):e26940.

3.	Yu H, Du R, Wang M, Yu F, Yang J, Jiao L, Wang Z, Liu H, Wu P, Bärnighausen T *et al*: **Attitudes Toward the Global Allocation of Chinese COVID-19 Vaccines: Cross-sectional Online Survey of Adults Living in China**. *JMIR Public Health Surveill* 2022, **8**(6):e33484.

4.	Xiao L, Parascandola M, Wang C, Jiang Y: **Perception and Current Use of E-cigarettes Among Youth in China**. *Nicotine Tob Res* 2019, **21**(10):1401–1407.

5.	Yoong SL, Hall A, Leonard A, McCrabb S, Wiggers J, Tursan d'Espaignet E, Stockings E, Gouda H, Fayokun R, Commar A *et al*: **Prevalence of electronic nicotine delivery systems and electronic non-nicotine delivery systems in children and adolescents: a systematic review and meta-analysis**. *Lancet Public Health* 2021, **6**(9):e661–e673.

6.	Craig CL, Marshall AL, Sjöström M, Bauman AE, Booth ML, Ainsworth BE, Pratt M, Ekelund U, Yngve A, Sallis JF *et al*: **International physical activity questionnaire: 12-country reliability and validity**. *Medicine and science in sports and exercise* 2003, **35**(8):1381–1395.

7.	Salmon J, Campbell KJ, Crawford DA: **Television viewing habits associated with obesity risk factors: a survey of Melbourne schoolchildren**. *Med J Aust* 2006, **184**(2):64–67.

8.	Paruthi S, Brooks LJ, D'Ambrosio C, Hall WA, Kotagal S, Lloyd RM, Malow BA, Maski K, Nichols C, Quan SF *et al*: **Consensus Statement of the American Academy of Sleep Medicine on the Recommended Amount of Sleep for Healthy Children: Methodology and Discussion**. *J Clin Sleep Med* 2016, **12**(11):1549–1561.

9.	Bischops AC, Radev ST, Köthe U, Chen S, Geldsetzer P, Sarker M, Su TT, Mohamed FA, Darwish N, Ahmad NA *et al*: **Data Resource Profile: The Global School-based Student Health Survey—behavioural risk and protective factors among adolescents**. *International Journal of Epidemiology* 2022, **52**(2):e102–e109.

10.	Sussman S, McCuller WJ, Dent CW: **The associations of social self-control, personality disorders, and demographics with drug use among high-risk youth**. *Addict Behav* 2003, **28**(6):1159–1166.

11.	Chen. F, Bi. C, Han. M: **The Reliability and Validity of the Chinese Version of the Revised-Positive version of Rosenberg Self-Esteem Scale**. *Advances in Psychology* 2015, **05**:531–535.

12.	Zhang J, Schwarzer R: **Measuring optimistic self-beliefs: A Chinese adaptation of the General Self-Efficacy Scale**. *Psychologia: An International Journal of Psychology in the Orient* 1995, **38**:174–181.

13.	Lu W, Bian Q, Wang W, Wu X, Wang Z, Zhao M: **Chinese version of the Perceived Stress Scale-10: A psychometric study in Chinese university students**. *PLoS One* 2017, **12**(12):e0189543.

14.	Wang W, Bian Q, Zhao Y, Li X, Wang W, Du J, Zhang G, Zhou Q, Zhao M: **Reliability and validity of the Chinese version of the Patient Health Questionnaire (PHQ-9) in the general population**. *Gen Hosp Psychiatry* 2014, **36**(5):539–544.

15.	Iacobucci D, Schneider MJ, Popovich DL, Bakamitsos GA: **Mean centering, multicollinearity, and moderators in multiple regression: The reconciliation redux**. *Behavior Research Methods* 2017, **49**(1):403–404.

16.	Ecija C, Gutiérrez L, Catalá P, Peñacoba C: **How to Promote Walking in Women with Fibromyalgia: A Look at Catastrophizing, Goal Conflict, and Avoidance from a Self-Emotional Regulatory Perspective**. *European Journal of Investigation in Health, Psychology and Education* 2024, **14**(8):2126–2139.

17.	Shallcross L, Burke D, Abbott O, Donaldson A, Hallatt G, Hayward A, Hopkins S, Krutikov M, Sharp K, Wardman L *et al*: **Factors associated with SARS-CoV-2 infection and outbreaks in long-term care facilities in England: a national cross-sectional survey**. *Lancet Healthy Longev* 2021, **2**(3):e129–e142.
